# Long non-coding RNA small nucleolar RNA host gene 12 (SNHG12) promotes tumorigenesis and metastasis by targeting miR-199a/b-5p in hepatocellular carcinoma

**DOI:** 10.1186/s13046-016-0486-9

**Published:** 2017-01-10

**Authors:** Tian Lan, Weijie Ma, Zhenfei Hong, Long Wu, Xi Chen, Yufeng Yuan

**Affiliations:** Department of Hepatobiliary surgery, Zhongnan Hospital of Wuhan University, No. 169 of Donghu Road, Wuhan, Hubei 430071 People’s Republic of China

**Keywords:** Hepatocellular carcinoma, SNHG12, miR-199a/b-5p, MLK3, ceRNA, Sponge

## Abstract

**Background:**

Hepatocellular carcinoma (HCC) is third leading cause of cancer-related death globally. Evidence suggest that long non-coding RNAs (lncRNAs) have emerged as key regulators of tumorigenesis and metastasis in HCC. In this study, we investigated the functional significance of SNHG12 and explored whether SNHG12 can directly interact with miR-199a/b-5p in the progression of HCC.

**Methods:**

We determined the expression level of SNHG12 in HCC tissues with quantitative real-time PCR and then studied its clinical significance. The binding site between SNHG12 and miR-199a/b-5p was confirmed using dual luciferase assay and RNA immunoprecipitation (RIP) assay. SNHG12 was silenced through the siRNA transfection to determine whether SNHG12-siRNA is able to affect cell proliferation, invasion and metastasis.

**Results:**

SNHG12 was significantly higher in the HCC tissues than that in the adjacent normal tissues. There were direct interactions between miR-199a/b-5p and the binding site of SNHG12. SNHG12 functioned as an endogenous sponge for miR-199a/b-5p to regulate the expression of MLK3 and affect the NF-κB pathway.

**Conclusion:**

SNHG12 may serve as a valuable biomarker and a potential therapeutic target for HCC.

## Background

Hepatocellular carcinoma (HCC) has been deemed the fifth most common type of malignant tumor. It has been estimated that about 500,000 to 1,000,000 new cases are diagnosed as HCC annually which has the third mortality rate among cancers [1]. Invasion, metastasis and postoperative recurrence are regarded as the main causes of death in patients with HCC. More seriously, HCC is frequently diagnosed at an advanced stage and therefore has a high lethality [2]. The molecular mechanism underlying HCC carcinogenesis has not been clearly elucidated. The development and progression of HCC are often described as a multistage process involving a series of genetic alterations that play the crucial roles in the malignant transformation of the hepatocytes [3]. Currently, an increasing number of studies reported the role of non-coding RNAs (ncRNAs) in the carcinogenesis of HCC, including micro-RNAs (miRNAs), long non-coding RNA (lncRNAs) and small nucleolar RNAs (snoRNAs) [4–6].

Long non-coding RNAs (lncRNAs) are a class of RNA molecule that are over 200 nucleotides in length and do not code for proteins but bear the ability to regulate the gene expression [4]. Accumulating evidence showed that lncRNAs have been identified to affect multiple biological regulatory processes, including development, differentiation and carcinogenesis [7, 8]. Additionally, the dysregulation of lncRNAs may be associated with the development and metastasis of various types of cancer [9]. For instance, the lncRNA metastasis-associated lung adenocarcinoma transcript 1 (MALAT1) was found to be overexpressed in numerous kinds of cancer cells, to regulate the cell proliferation and inhibit the cell cycle [10]. A recent study demonstrated that the lncRNA small nucleolar RNA host gene 12 (SNHG12) promotes cell proliferation and migration by upregulating AMOT gene expression in human osteosarcoma [11]. However, the functional role and specific mechanism of SNHG12 in HCC remain completely unclear. It is reported that all RNA transcripts bearing miRNA-binding sites can interact and regulate each other’s expression levels by specifically competing for shared miRNAs, acting as competing endogenous RNAs (ceRNAs) [12]. Emerging evidence illustrated that this ceRNA regulation mode was confirmed in the various cancers [13, 14], indicating that there are interactions between the lncRNAs and miRNAs in the tumorigenesis [15, 16].

In the present study, we investigated the relationship between SNHG12 and HCC, and found that SNHG12 was significantly overexpressed in HCC tissues as compared with adjacent normal tissues, and this overexpression of SNHG12 was associated with substantially declined survival of HCC patients. Furthermore, functional analysis and bioinformatics prediction indicated SNHG12 inhibited both cell proliferation and tumorigenicity of HCC cells, possible by targeting miR-199a/b-5p and then effects on NF-κB pathway.

## Methods

### Tissue specimens and cell lines

The paired HCC samples and the adjacent non-tumor tissues were collected following curative surgical resection from 48 patients with HCC in the Zhongnan Hospital of Wuhan University between May 2011 and May 2013. The present study was approved by the Hospital’s Protection of Human Subjects Committee and written informed consent was obtained from all patients. Based on their medical documents, we conducted a 48-month follow up survival survey. Overall survival (OS) was defined as the interval between resection and death or the last follow-up visit. Recurrence free survival (RFS) was defined as the interval between treatment and the first diagnosis of metastasis or recurrence. Curative resection was defined as the removal of all recognizable tumor tissue with a clear microscopic margin. Patients were not treated using any preoperative therapy, such as transcatheter arterial chemoembolization, radiofrequency ablation or percutaneous ethanol injection. SK-Hep1 cell line included in this study was purchased from the Cell Bank of Type Culture Collection (CBTCC, Chinese Academy of Sciences, Shanghai, China) and cultured in DMEM/high glucose medium (GE Healthcare Life Sciences HyClone Laboratories, Logan, USA) supplemented with 10% fetal bovine serum (FBS; Gibco; Thermo Fisher Scientific, Inc., Waltham, MA, USA) and 1% penicillin and streptomycin in a humidified atmosphere of 5% CO2 at 37 °C.

### In situ hybridization (ISH)

ISH was used to detect SNHG12 in 48 paired of HCC samples. A digoxigenin-UTP-labeled antisense RNA probe (Beijing View Solid Biotechnology) was derived from 28 to 257 nt of SNHG12 by in vitro transcription using the DIG RNA Labeling kit (Roche Diagnostics, Indianapolis, IN, USA). The digoxigenin-UTP labeled sense RNA probe derived from 28 to 257 nt of SNHG12 was used as a negative control. ISH was performed using the ISH kit (Boster Bio-Engineering Company, Wuhan, China).

### RNA extraction and quantitative real-time PCR

Total RNA was extracted from clinical HCC specimens and human HCC cell lines in Trizol reagent kit (Invitrogen, Carlsbad, CA, USA), according to the manufacturer’s instructions. RNA concentration was measured by using a NanoDrop ND-2000 spectrophotometer (Life Technologies). Reverse transcription was conducted with random primers using the First Strand cDNA synthesis kit (Takara, Otsu, Shiga, Japan). Real-time quantitative PCR was performed with an iQ SYBR-Green Supermix (Bio-Rad, California, USA). All reactions were run in triplicate on the iCycler IQ multi-color Detection System (Bio-Rad, California, USA). The amplification profile was denatured at 95 °C for 10 min, followed by 45 cycles of denaturation at 95 °C for 20 s, annealing at 60 °C for 20 s and extension at 72 °C for 20 s. The comparative cycle threshold (CT) method was applied to quantify the expression levels of mRNA. The relative amount was calculated using the equation 2 − ΔCT where ΔCT = (CT interest mRNA – CT U6). All qPCR reactions were performed in duplicate. Primers used in this study are listed in Table 1.

**Table 1:** Primer sequences for qPCR

Gene	Primer	Sequence
SNHG12	Forward primer	5′-TCTGGTGATCGAGGACTTCC-3′
	Reverse primer	5′-ACCTCCTCAGTATCACACACT-3′
miR-199a-5p	Stem-loop primer	5′-ctcaactggtgtcgtggagtcggcaattcagttgagGAACAGG-3′
	Forward primer	5′-acactccagctgggCCCAGT-3′
miR-199b-5p	Stem-loop primer	5′-ctcaactggtgtcgtggagtcggcaattcagttgagGAACAGA-3′
	Forward primer	5′-acactccagctgggCCCAGT-3′
	Reverse primer	5′-TGGTGTCGTGGAGTCG-3′
U6	Forward primer	5′-GCTTCGGCAGCACATATACTAAAAT-3′
	Reverse primer	5′-CGCTTCACGAATTTGCGTGTCAT-3′
SNORA44	Forward primer	5′-CTCCAACTGCATGCAAGAGC-3′
	Reverse primer	5′-ATAGGAAAGCTGAGTGGCAGC-3′
SNORA61	Forward primer	5′-CACTGGTCTTGGTGGTCGTAA-3′
	Reverse primer	5′-ACCTGTCTGAAACTAGCCCAC-3′
SNORA16A	Forward primer	5′-CTGCTGTGGTCAAAAAGGAGC-3′
	Reverse primer	5′-TCAGTTACAACAAACAGAACGGC-3′
SNORD99	Forward primer	5′-ACTGGTCCAGGATGAAACCTA-3′
	Reverse primer	5′-GGTCTCAGTCCCATATCCGC-3′

### RNA interference and cell transfection

The small interfering RNA (siRNA) targeting SNHG12 was obtained from Sigma-Aldrich (Sigma, USA), and the miR-199a/b-5p mimic was synthesized by Genepharma (Shanghai, China). SK-Hep1 cells were transfected with 50 nM miR-199a/b-5p mimic using Lipofectamine 2000 (Invitrogen Co., Carlsbad, CA, USA) and transfected with 100 nM SNHG12-siRNA using GenMute (SignaGen Laboratories, Rockville, MD, USA) according to the manufacturer’s protocol. After transfection, cells were harvested for qPCR analysis or western blot.

### Protein extraction and western blot

Total proteins were extracted with RIPA Lysate Buffer (Beyotime Institute of Biotechnology, Shanghai, China), separated by 10% SDS-PAGE and transferred onto PVDF membranes. Subsequently, the PVDF membrane was blocked with 5% skim milk powder in TBST (10 mM Tris–HCl, pH 7.5, 150 mM NaCl, and 0.1% Tween-20) at room temperature for 2 h. Then, the primary antibodies were added and incubated overnight at 4 °C. After washing with TBST buffer, the HRP-labeled antibody (Proteintech Group, Inc., Chicago, IL, USA; 1:4000 dilution) was added and the membrane was incubated at RT for 2 h. The proteins were visualized by autoradiography using the ECL chemiluminescence reagent (Bio-Rad, California, USA). The relative expression of interest protein was represented as the grayscale ratio of the protein to GAPDH and the results were analyzed by GraphPad Prism 5.0 software. The information of antibodies used in this study was presented in Table 2.

**Table 2:** Antibody information used in this study

Antibodies	Type	Raised and targeted species	Dilution	Catalogue number	Supplier
anti-GAPDH	polyclonal	rabbit anti-human	1/4000	10494-1-AP	Proteintech Group, Inc., Chicago, IL, USA
anti-MLK3	monoclonal	rabbit anti-human	1/5000	ab51068	Abcam, Cambridge, MA, USA
anti-IκB-α	monoclonal	rabbit anti-human	1/2000	ab32518	Abcam, Cambridge, MA, USA
anti-pIκB-α	monoclonal	rabbit anti-human	1/2000	ab92700	Abcam, Cambridge, MA, USA
anti-NF-κB/p65	polyclonal	rabbit anti-human	1/2000	ab16502	Abcam, Cambridge, MA, USA
anti-pNF-κB/p65	polyclonal	rabbit anti-human	1/2000	ab86299	Abcam, Cambridge, MA, USA
anti-Erk1/2	monoclonal	rabbit anti-human	1/1000	4695S	Cell Signaling Technology, Danvers, MA, USA
anti-pErk1/2	monoclonal	rabbit anti-human	1/1000	4376S	Cell Signaling Technology, Danvers, MA, USA
anti-Ago2	polyclonal	rabbit anti-human		ab32381	Abcam, Cambridge, MA, USA
HRP-conjugated secondary antibody		goat anti-mouse	1/4000	SA00001-1	Proteintech Group, Inc., Chicago, IL, USA
		goat anti-rabbit	1/4000	SA00001-2	Proteintech Group, Inc., Chicago, IL, USA

### Cell proliferation assay

A total of 3 × 10^4^ cells were seeded in each well of a 96-well plate, and incubated for 24 h. Ten Microliter WST-8 from the CCK-8 Kit (Boster, Wuhan, China) was then added to each well. After incubation at 37 °C in 5% CO2 for 12, 24, 48, 72 and 96 h, the absorbance of each sample was measured at a wavelength of 450 nm by using the Thermo Plate microplate reader (Rayto Life and Analytical Science Co. Ltd., Germany).

### Flow cytometry analysis

Cell apoptosis was tested by two-color immunofluorescence staining in the flow cytometric analysis. Cells were trypsinized without EDTA followed by two washing steps with phosphate buffered saline. 2 × 10^5^ cells per well were then incubated with 5 μl of FITC Annexin V (BD Pharmingen) and 5 μl of propidium iodide (BD Pharmingen) in 400 μl of 1 × binding buffer for 15 min at room temperature. Data were analyzed by flow cytometry (FACScan®; BD Biosciences, San Jose, CA) equipped with CellQuest software (BD Biosciences).

### Cell invasion assay

Transwell chambers were precoated with 200 μg/ml Matrigel (BD Biosciences, San Jose, CA, USA) and incubated overnight. SK-Hep1 cells were cultured in serum-free medium in the upper chambers of a Transwell (Corning, NY, USA) plate, which are separated from the lower chambers with permeable 8.0 μm polycarbonate membranes. The lower chamber was filled with 500 μL DMEM containing 10% FBS. After incubation for 24 h, the cells remaining on the upper membrane were carefully removed, the others that had invaded through the membrane were fixed with 4% paraformaldehyde and stained with 0.1% crystal violet. The number of stained cells were manually counted under a phase contrast microscope from three different visual fields of each filters.

### Cell migration assay

Cells were cultured in 6-well plates (seeding density 1 × 10^6^ cells/well). Confluent cell monolayers was disrupted by standardized wound scratching using a sterile 10 μL pipette tip and incubated in serum-free medium. The size of the wound was measured after 24 h of wound formation and photographed by using a phase contrast microscope.

### Construction of the recombinant plasmid

The full-length SNHG12 was amplified by PCR, and the PCR products were digested and ligated into pMir-Reporter plasmid (Ambion, Life Technologies, Carlsbad, CA, USA). The miR-199a/b-5p binding site mutations were generated using a QuikChange Multi Site-Directed Mutagenesis Kit (Stratagene, La Jolla, CA, USA). The insertion and mutation were verified using sequencing.

### Dual luciferase assay

Cells were cultured overnight until 60–70% confluence. Next, cells were co-transfected with pMir-Reporter-SNHG12-WT or pMir-Reporter-SNHG12-MUT and miR-199a/b-5p mimic. Lipofectamine 2000 (Invitrogen Co., Carlsbad, CA, USA) was used according to the manufacturer’s instructions. After 48 h, cells were harvested for luciferase detection using the dual-luciferase reporter gene assay system (Promega, Madison, WI, USA). All values were obtained from at least three independent repetitions of the transfection.

### RNA immunoprecipitation (RIP) assay

RIP assay was performed using the Magna RIP^TM^ RNA-Binding Protein Immunoprecipitation Kit (Millipore, Bedford, MA, USA) according to the manufacturer’s protocol. Cells at 80–90% confluence were collected and were lysed using RIP lysis buffer. Next, the cell extracts were incubated with RIP buffer containing magnetic beads conjugated with human anti-Ago2 antibody or negative control IgG. The samples were incubated with Proteinase K to digest the protein and subsequently the precipitated RNA was obtained. The purified RNA was finally used for qPCR analysis of SNHG12 and miR-199a/b-5p.

### ChIRP assay

17 oligonucleotide probes corresponding to the SNHG12 transcript were produced by using the Stellaris Probe Designer (https://www.biosearchtech.com/stellarisdesigner/). The oligonucleotide sequences (Presented in Table 3) were synthesized with an 18-atom spacer followed by a biotin tag located at the 3′ end, according to the manufacturer (IDT, Coralville, Iowa). The autopod regions of the E 11 limbs were dissected and digested in PBS containing 0.1% trypsin and 0.1% collagenase for 15 mins. Then, cell lysis and streptavidin bead precipitation were completed utilizing a Bioruptor instrument (Diagenode). Cells with SNHG12-WT and SNHG12-MUT were used in parallel assays. The precipitated RNA was identified and quantified by using RT-qPCR. The relative amount was calculated using the equation 2 − ΔCT where ΔCT = (CT miR-199a/b-5p – CT SNHG12). All qPCR reactions were performed in duplicate.

**Table 3:** LncRNA-SNHG12 ChIRP probe sets

Probe	
1	5′-gaggaaaaacccggcgagtg/iSp18//3Bio/-3′
2	5′-acattcaccaccatctcgag/iSp18//3Bio/-3′
3	5′-cagtccgaagcgagagaagg/iSp18//3Bio/-3′
4	5′-cttgatgggaccgttttatc/iSp18//3Bio/-3′
5	5′-caatagctggtgtgcttttt/iSp18//3Bio/-3′
6	5′-taagtcagtcatcctgtagg/iSp18//3Bio/-3′
7	5′-cttcatctgcttaagtacgc/iSp18//3Bio/-3′
8	5′-caccttctgtcatcttaaga/iSp18//3Bio/-3′
9	5′-agtcctcgatcaccagaaaa/iSp18//3Bio/-3′
10	5′-acacactgccaggaaacttg/iSp18//3Bio/-3′
11	5′-aaacaagctcacctcctcag/iSp18//3Bio/-3′
12	5′-gaatcttaaagcacagctcc/iSp18//3Bio/-3′
13	5′-gaggacagctttacatgtaa/iSp18//3Bio/-3′
14	5′-ccataggtccatagtcacaa/iSp18//3Bio/-3′
15	5′-tcccatagagattgtcccaa/iSp18//3Bio/-3′
16	5′-tctacctaaaatgaccgggg/iSp18//3Bio/-3′
17	5′-ctctgaagttcagtagcaca/iSp18//3Bio/-3′

### Statistical analysis

All experiments were performed in triplicate and data were presented as mean ± SD. All statistical analyses were performed with SPSS version 22.0 software (IBM, Chicago, IL, USA). The expression level of SNHG12 in HCC samples was compared with adjacent normal tissues utilizing the paired sample t-test. The association between SNHG12 expression and clinicopathological variables was evaluated using the chi-square test or Fisher’s exact test. The Kaplan-Meier test was used to estimate OS and RFS. The expression differences between groups, the expression changes after transfection, cell viability and cell invasion assays were analyzed using independent samples t-test or one-way analysis of variance (ANOVA). A value of *P* < 0.05 was considered to be statistically significant.

## Results

### SNHG12 was strongly overexpressed in HCC tissues and significantly associated with clinicopathological variables and prognosis after surgery

**Fig. 1:**
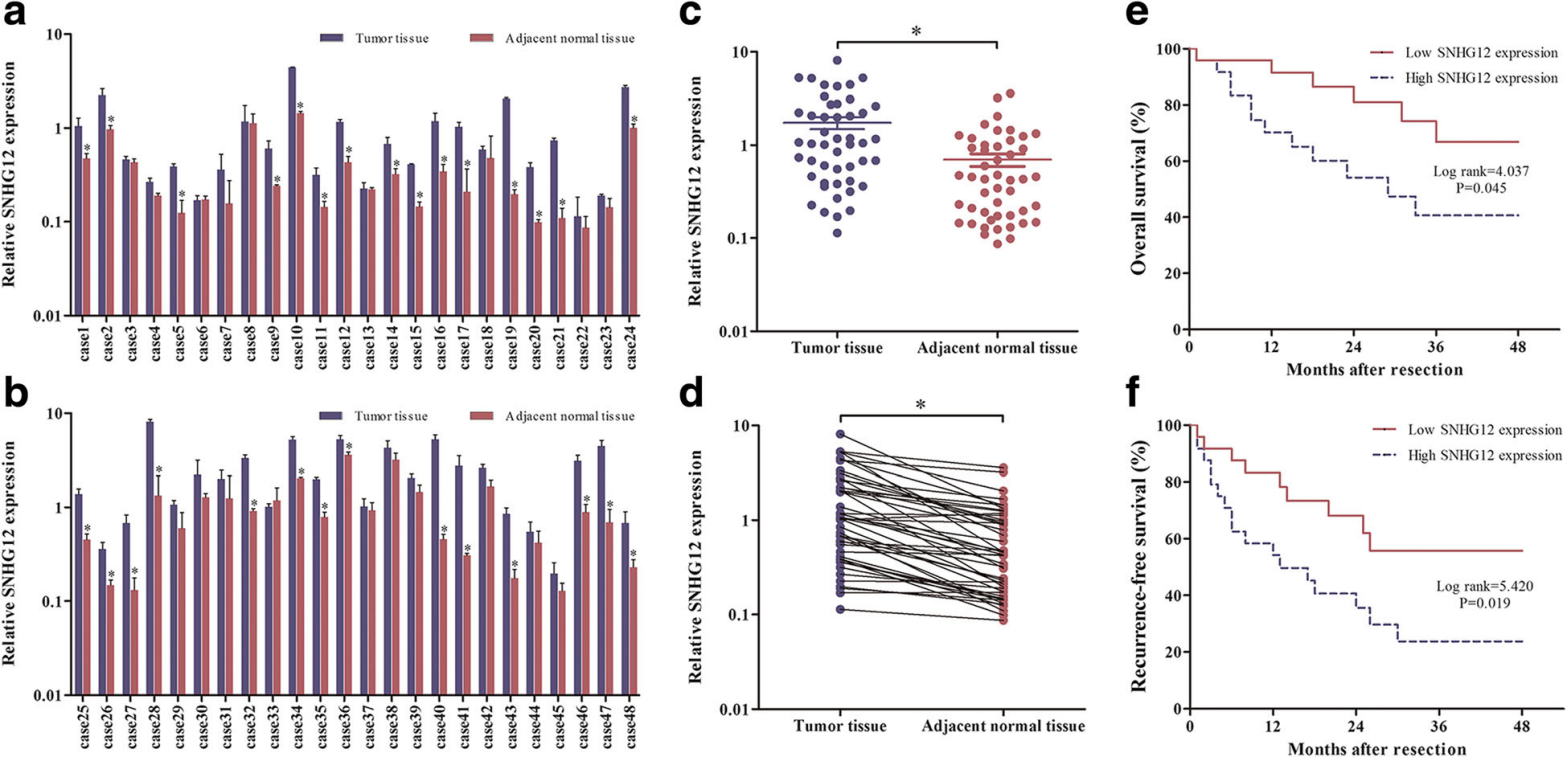
SNHG12 was overexpressed in HCC tissues and was associated with prognosis after surgery. **a** and **b** Relative SNHG12 expression in HCC tissues (*n* = 48) compared with adjacent normal tissues (*n* = 48). SNHG12 expression was examined by qPCR and normalized to U6 expression. Results were presented as ΔCT in tumor tissues relative to normal tissues. **c** and **d** SNHG12 expression was higher in HCC tissues compared with the paired adjacent normal tissues. Data are presented as the mean ± standard error of the mean (*n* = 3). Kaplan-Meier analysis of (**e**) overall survival and (**f**) recurrence-free survival based on SNHG12 expression levels in 48 patients with HCC. The median level of SNHG12 was used as the cut off. Patients with HCC were divided into high SNHG12 expression group and low SNHG12 expression group. *P < 0.05

To determine whether there was a difference of SNHG12 expression between HCC tissues and the adjacent normal tissues, we detected the SNHG12 expression in 48 paired specimens utilizing the qPCR (Fig. 1a and b). The expression of SNHG12 was significantly overexpressed in cancerous tissues (*P* < 0.001; Fig. 1c and d). Furthermore, in order to assess the correlation between SNHG12 expression and clinicopathological variables, patients were divided into the high expression group (*n* = 24) and the low expression group (*n* = 24), based on the median expression level of all tumor tissues. Specifically, as presented in Table 4, SNHG12 expression levels in HCC were significantly associated with tumor size (*P* < 0.05), vascular invasion (*P* < 0.05), and TNM stage (*P* < 0.05). However, SNHG12 expression was not correlated with other clinicopathological variables such as gender, age, serum AFP, liver cirrhosis, PVTT and differentiation (*P* > 0.05). Besides, HCC patients with high SNHG12 expression have significantly shorter overall survival (*P* = 0.045; Fig. 1e) and statistically higher recurrence rate (*P* = 0.019; Fig. 1f) than those with low expression of SNHG12. These findings illustrated that the overexpression of SNHG12 may be associated with tumorigenesis and poor prognosis in patients with HCC.

**Table 4:** Correlation between SNHG12 expression and clinicopathological variables of HCC patients

Variable	Total no. (*n* = 48)	Relative SNHG12 expression		*P* value
		High (*n* = 24)	Low (*n* = 24)	
Gender				
Male	37	19	18	0.7313
Female	11	5	6	
Age (years)				
<60	23	10	13	0.3861
≥60	25	14	11	
Tumor size (cm)				
<5	20	6	14	0.0192*
≥5	28	18	10	
Serum AFP (ng/ml)				
<200	9	4	5	1.0000
≥200	39	20	19	
Liver cirrhosis				
Present	25	15	10	0.1486
Absent	23	9	14	
Vascular invasion				
Present	23	16	7	0.0093*
Absent	25	8	17	
PVTT				
Present	8	5	3	0.7008
Absent	40	19	21	
Differentiation				
Well	7	4	3	0.6327
Moderate	33	15	18	
Poor	8	5	3	
TNM stage				
I–II	22	7	15	0.0415*
III–IV	26	17	9	

*Abbreviation*: *AFP* α-fetoprotein, *PVTT*, portal vein tumor thrombus, *TNM* tumor-node-metastasis

* *P* < 0.05

### Knockdown of SNHG12 suppressed HCC cell proliferation and induces cell apoptosis in vitro

**Fig. 2:**
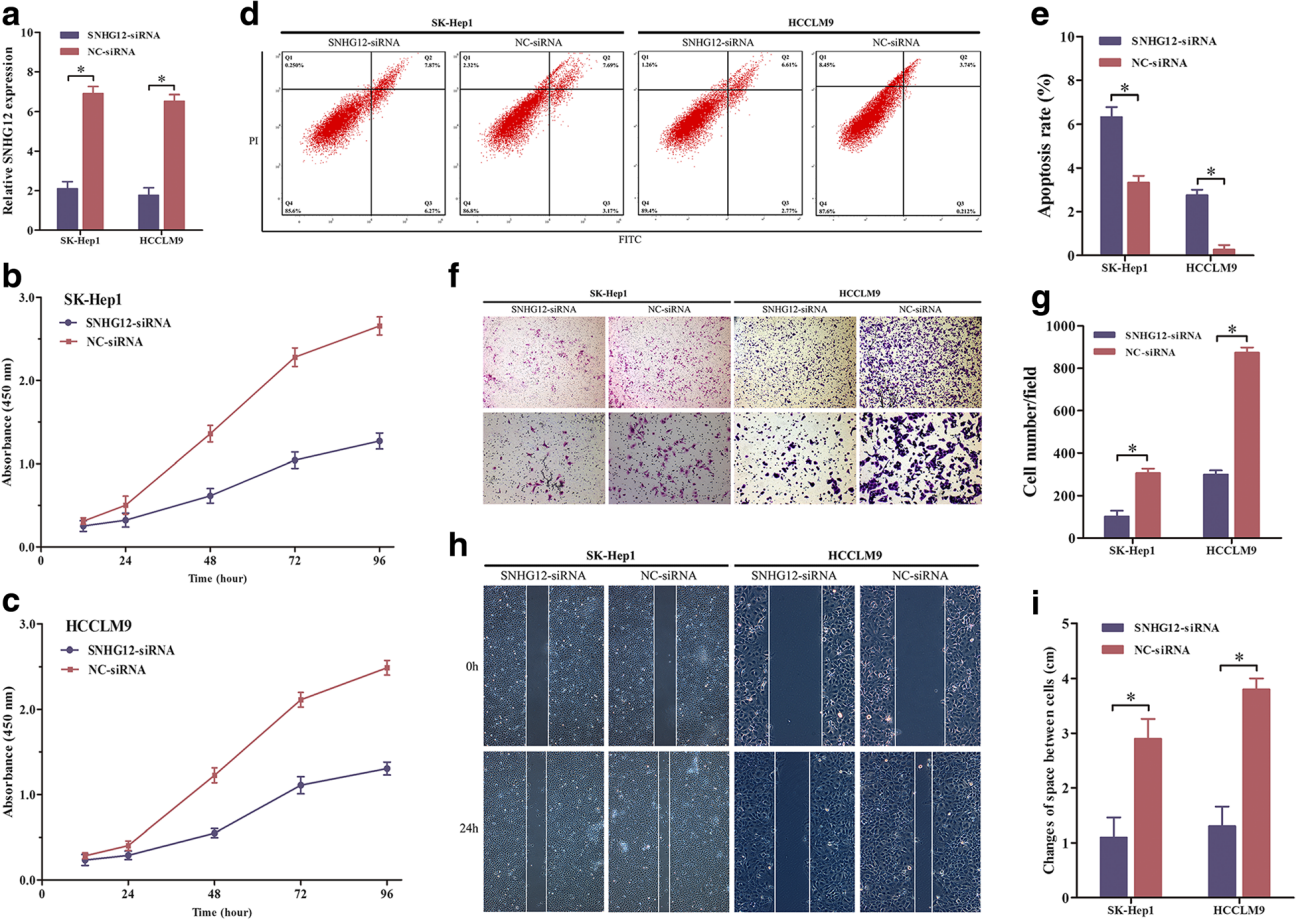
The functional analysis of SNHG12 in HCC cells. **a** The SNHG12 expression level was determined by qPCR when SK-Hep1 and HCCLM9 cells were transfected with SNHG12-siRNA. **b** CCK-8 assay was applied to detect the proliferation of SK-Hep1 cells. **c** CCK-8 assay was applied to detect the proliferation of HCCLM9 cells. **d** and **e** Flow cytometry assays were performed to analyze the apoptosis of SK-Hep1 and HCCLM9 cells after treatment with SNHG12-siRNA and stained with apoptosis markers (FITC-Annexin V and PI). **f** and **g** The ability of cancer cell invasion was measured by using transwell assay when SNHG12 was downregulated in SK-Hep1 and HCCLM9 cells [original magnification, ×250 and × 100]. **h** and **i** The ability of cancer cell migration was measured by using wound-healing assay when SNHG12 was downregulated in SK-Hep1 and HCCLM9 cells [original magnification, ×100]. Data represented the mean ± SD from three independent experiments. *P < 0.05; NS: no significance

To investigate the role of SNHG12 on HCC cell proliferation, SNHG12-siRNA was transfected into SK-Hep1 and HCCLM9 cells. Next, both two cell lines were divided into two treatment groups: the SNHG12-siRNA transfected group and the NC-siRNA group. The knockdown effect of SNHG12 was confirmed by qPCR assay which revealed that the SNHG12 expressions in SK-Hep1 and HCCLM9 cells of the SNHG12-siRNA transfected group were significantly decreased by approximately 70% (*P* < 0.05; Fig. 2a). Therefore, the interference ability of siRNA for SNHG12 was effective and specific. Then, the CCK-8 assay showed that the proliferation abilities of both SK-Hep1 and HCCLM9 cells presented statistical decline after knockdown of SNHG12 (*P* < 0.05; Fig. 2b and c). Subsequently, flow cytometry analysis was performed to further examine the effect of SNHG12 on the apoptosis of SK-Hep1 and HCCLM9 cells, indicating that the proportion of cells in early apoptosis witnessed a remarkable upward trend by the silencing of SNHG12 (*P* < 0.05; Fig. 2d and e).

### Effects of SNHG12 interfering on HCC cell invasion and migration in vitro

Cell invasion and migration assays were performed to determine how SNHG12 affected these characteristics in SK-Hep1 and HCCLM9 cells. The transwell chamber assay suggested that the numbers of SK-Hep1 and HCCLM9 cells that had passed through the polycarbonate membrane of the chamber were significantly reduced in the SNHG12-siRNA transfected group (*P* < 0.05; Fig. 2f and g). The wound-healing assay was conducted to investigate the migration abilities of SK-Hep1 and HCCLM9 cells in these two treatment groups. It was determined that the migration ability of cells in the SNHG12-siRNA transfected group presented a significantly decrease, 24 h after wounding (*P* < 0.05; Fig. 2h and i). These findings demonstrated that the downregulation of SNHG12 weakened the migration and invasion abilities of SK-Hep1 and HCCLM9 cells.

### SNHG12 regulated HCC tumorigenesis and metastasis by targeting miR-199a/b-5p and affected the NF-κB pathway

**Fig. 3:**
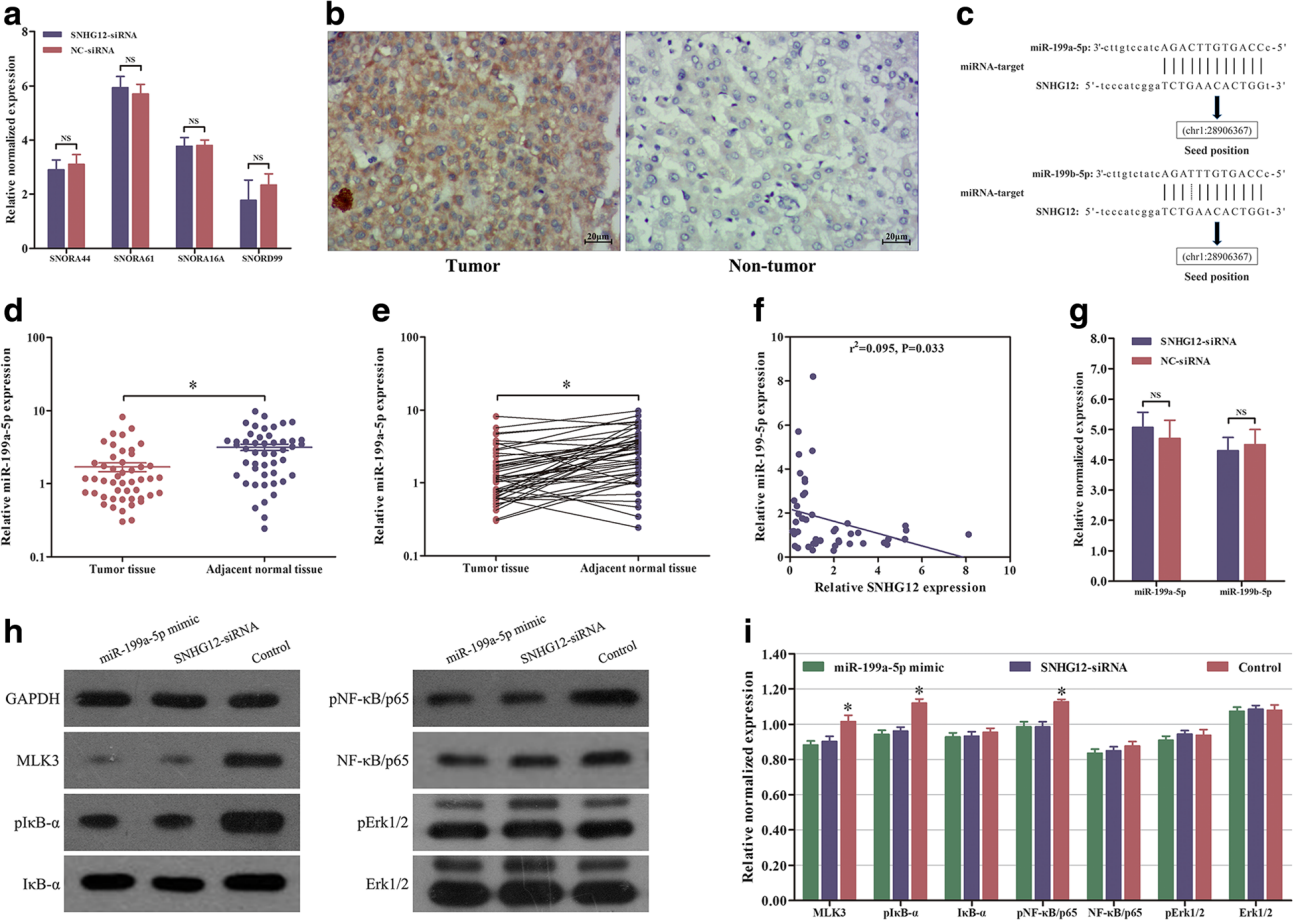
SNHG12 regulated the expression of miR-199a/b-5p target gene and affected the NF-κB pathway. **a** The expressions of related snoRNAs were determined by qPCR when SK-Hep1 cells were transfected with SNHG12-siRNA. **b** Representative images of SNHG12 expression in human HCC tissues and adjacent non-tumor tissues by ISH assays. **c** Predict binding sites between SNHG12 and miR-199a/b-5p. **d** and **e** The expression of miR-199a-5p was lower in HCC tissues compared with the paired adjacent normal tissues by qPCR. **f** Bivariate correlation analysis of the negative association between SNHG12 expression level and miR-199a-5p expression level in 48 pairs of HCC tissues. **g** The miR-199a/b-5p expression level was determined by qPCR when SK-Hep1 cells were transfected with SNHG12-siRNA. **h** and **i** Western blot analysis of key molecules in NF-κB pathway after SNHG12 knockdown or miR-199a-5p overexpression in SK-Hep1 cells. Relative protein expression was identified and normalized to GAPDH. Data represented the mean ± SD from three independent experiments. *P < 0.05; NS: no significance

Since SNHG12 is the host gene of four small nucleolar RNAs (snoRNAs): SNORA44, SNORA61, SNORA16A and SNORD99, we hypothesized that SNHG12 might exert its oncogenic function via these snoRNAs, and thus we detected the effect of SNHG12 on their expressions by using RT-qPCR. The data showed that knocking down SNHG12 did not impact the snoRNAs expression (Fig. 3a). To address the regulatory mechanism of SNHG12 in HCC cell proliferation and invasion, we firstly conducted the ISH assay to determine its subcellular localization. The results showed that SNHG12 was primarily located in the cytoplasm (Fig. 3b). Next, bioinformatics prediction of miRcode (http://www.mircode.org/) software suggested that there was putative binding sites between SNHG12 and miR-199a/b-5p (Fig. 3c). As miR-199a-5p and miR-199b-5p have the same seed sequence and their functions in tumorigenesis are almost identical [17–19], we selected miR-199a-5p as the study object to analyze whether its expression was regulated by SNHG12. By performing qPCR in all the 48 paired HCC samples and the adjacent normal tissues, we verified significantly lower miR-199a-5p expression in the tumor tissues (Fig. 3d and e). Intriguingly, an inverse correlation was found between SNHG12 and miR-199a-5p expression levels in 48 pairs of HCC tissues (Fig. 3f). Subsequently, in order to verify the influence of SNHG12 on miR-199a/b-5p expression, SK-Hep1 cells were transfected with SNHG12-siRNA. The cells with SNHG12 knockdown did not present significantly changed miR-199a/b-5p expression (Fig. 3g), which suggested that SNHG12 could not impact the expression of miR-199a/b-5p in HCC. Recent study has shown that decrement of miR-199a-5p contributes to the tumorigenesis by directly regulating MLK3/IkB/NF-κB pathway [20]. Likewise, MLK3 was proved to be the target gene of miR-199b-5p and was directly regulated by miR-199b-5p [21]. According to the previous prediction and the ceRNA regulation mode, we speculated that SNHG12 was able to regulate the MLK3/IκB/NF-κB pathway. To confirm that, the key molecules in the NF-κB pathway (MLK3, IκB-α and NF-κB/p65) were detected by western blot. As shown in Fig. 3h and i, both elevated expression of miR-199a-5p and knockdown of SNHG12 suppressed the expression of MLK3 and the phosphorylation of IκB-α and NF-κB/p65 in SK-Hep1 cells. By contrast, the phosphorylation of Erk1/2, a key regulator of MAPK signaling pathway, was not affected. These results indicated that the MLK3/IκB/NF-κB pathway may be involved in tumorigenesis induced by SNHG12.

### SNHG12 directly interacted with miR-199a/b-5p and served as its sponge

**Fig. 4:**
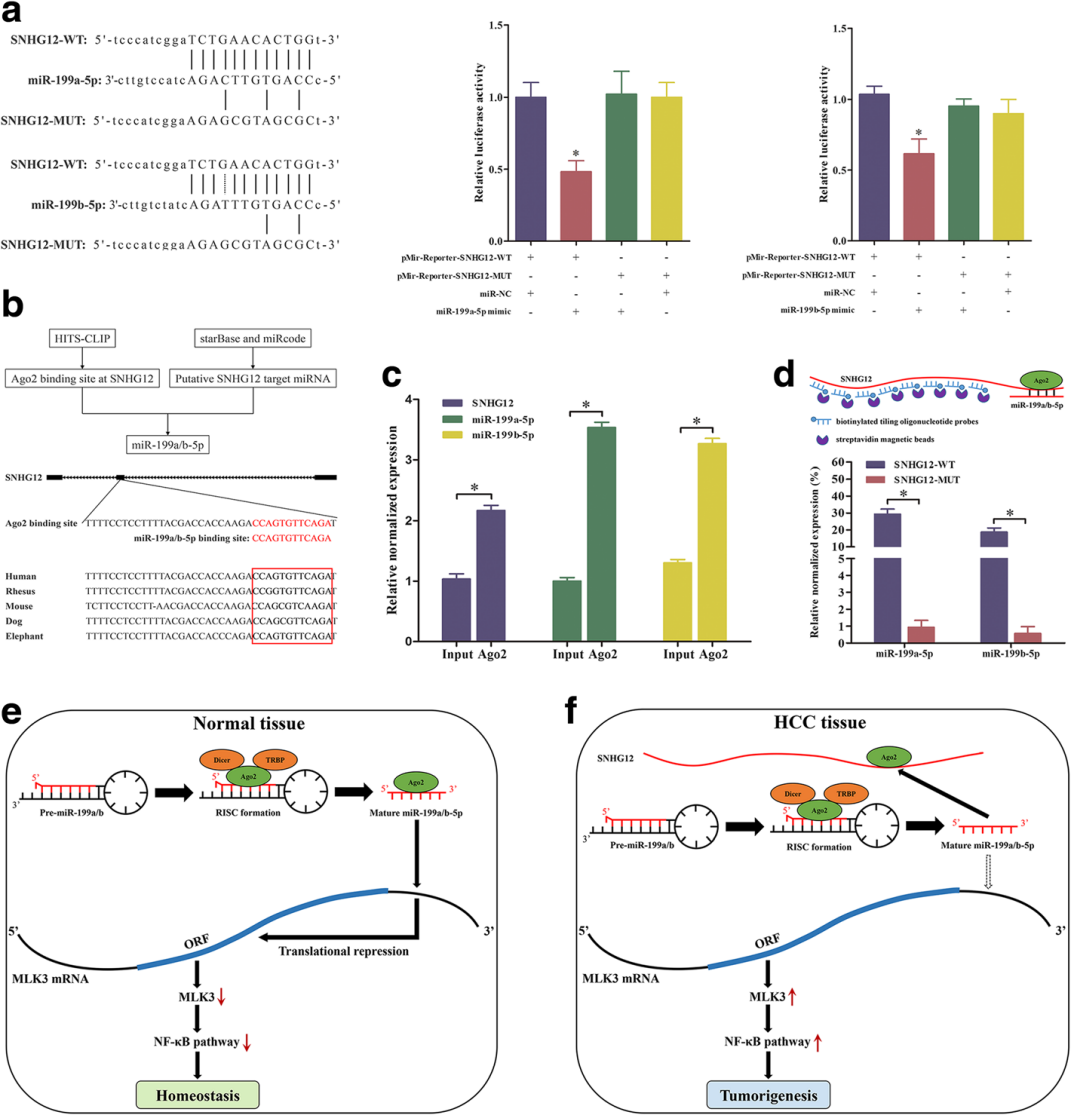
SNHG12 directly interacted with miR-199a/b-5p and served as a sponge. **a** Wild type and mutant SNHG12 sequences were cloned into pMir-Reporter vectors and co-transfected with miR-199a/b-5p mimic or miR-NC into SK-Hep1 cells. The relative luciferase activity was normalized with renilla luciferase activity. **b** Use of the StarBase and miRcode databases showed that the putative binding sites for both miR-199a/b-5p and the Ago2 protein at SNHG12 is highly conserved in human, rhesus, mouse, dog and elephant. **c** RNA immunoprecipitation with the anti-Ago2 antibody was used to assess endogenous Ago2 binding to RNA. The levels of SNHG12 and miR-199a/b-5p were detected by qPCR. **d** ChIRP assay followed by RT-qPCR to detect miR-199a/b-5p. **e** and **f** SNHG12 functioned as a sponge for miR-199a/b-5p and activated the NF-κB pathway which promoted the tumorigenesis in HCC. Data represented the mean ± SD from three independent experiments. *P < 0.05; NS: no significance

To determine whether there were direct interactions between SNHG12 and miR-199a/b-5p, the full-length SNHG12 was amplified by PCR, and the PCR products were digested and ligated into pMir-Reporter plasmid named pMir-Reporter-SNHG12-WT. Similarly, the mutant construct without the putative binding site was also generated and termed pMir-Reporter-SNHG12-MUT. The plasmids were respectively transfected into SK-Hep1 cells together with miR-199a/b-5p mimic or the negative control. The data revealed that luciferase activity of pMir-Reporter-SNHG12-WT was significantly reduced by miR-199a/b-5p mimic (Fig. 4a). In contrast, the luciferase activity of pMir-Reporter-SNHG12-MUT experienced no statistical changes (Fig. 4a). These findings indicated that there were interactions between miR-199a/b-5p and the binding sites of SNHG12. In addition, we found that the sites could also bind the Argonaute 2 (Ago2) protein, according to the prediction in StarBase v2.0 (http://starbase.sysu.edu.cn/mirLncRNA.php) (Fig. 4b). Previous study showed that miRNAs exert their gene silencing functions via RNA-induced silencing complex (RISC) containing Ago2 [22]. Emerging evidence demonstrated that lncRNAs might act as the sponge of miRNAs and function through binding the miRNAs and Ago2 [14]. Thus, we hypothesized that SNHG12 and miR-199a/b-5p might interact in the same way. To verify the speculation, RNA binding protein immunoprecipitation (RIP) assay was performed in SK-Hep1 cell extracts utilizing the antibody against Ago2. The levels of SNHG12 and miR-199a/b-5p were detected by qPCR. The results illustrated that both SNHG12 and miR-199a/b-5p were statistically enriched in Ago2 pellet relative to control IgG immunoprecipitates (Fig. 4c), indicating that Ago2 could directly bind the SNHG12 and miR-199a/b-5p in SK-Hep1 cells. Furthermore, we performed the ChIRP assays to demonstrate the direct interactions between SNHG12 and the miR-199a/b-5p. The data revealed that SNHG12-WT could be immunoprecipitated with miR-199a/b-5p. However, the SNHG12 with mutated binding site abolished the interaction (Fig. 4d), indicating that both miR-199a-5p and miR-199b-5p could directly bind to SNHG12 via a miRNA recognition site. Together, our results suggested that SNHG12 served as a sponge for miR-199a/b-5p and its oncogenic function greatly depended upon miR-199a/b-5p.

## Discussion

Growing study reported that ncRNAs have been identified to be dysregulated in a wide range of physiological and pathological processes, as well as malignant diseases [23, 24]. A number of studies have indicated the role of lncRNAs in cancer progression and predicts patients’ outcome [7, 10].

In this study, we systematically investigated potential role of oncogenic lncRNA SNHG12 and provided first evidence of SNHG12 dysregulation in HCC. Our results showed that the expression of SNHG12 in HCC tissues was significantly higher than that in adjacent normal tissues and it was remarkably associated with tumor size (*P* < 0.05), vascular invasion (*P* < 0.05), and TNM stage (*P* < 0.05). Additionally, based on the 48-month follow-up survival survey, our findings revealed that HCC patients with high SNHG12 expression have poorer OS and DFS than those with low expression of SNHG12 (*P* < 0.05, respectively), suggesting that SNHG12 could be regarded as a prognostic factor for HCC. Next, we determined the in vitro functional significance of SNHG12 in HCC cell lines via RNA interference. The data demonstrated that SNHG12 silencing inhibited cell proliferation by inducing cell apoptosis. Furthermore, the abilities of invasion and migration have been suppressed by the knockdown of SNHG12.

We observed an inverse correlation between SNHG12 and miR-199a-5p, which stimulated our interest to determine whether there was a ceRNA mechanism involved between SNHG12 and miR-199a/b-5p. To begin with, we performed the bioinformatics analysis to seek putative binding sites between them. Following that, it was proved that miR-199a/b-5p could bind the SNHG12 by conducting the dual luciferase assay. Most importantly, we found an endogenous interaction between SNHG12 and miR-199a/b-5p by utilizing co-immunoprecipitation with the Ago2 antibody in SK-Hep1 cells. Subsequently, ChIRP assays demonstrated that there were direct interactions between SNHG12 and the miR-199a/b-5p. Eventually, SNHG12 was able to modulate the expression of the miR-199a/b-5p target gene, MLK3, and thus affected the NF-κB pathway. Overall, these results demonstrated that SNHG12 could act as an endogenous sponge, or ceRNA, to regulate miR-199a/b-5p availability for the target gene. Mixed-lineage protein kinase 3 (MLK3) is a member of the mitogen-activated protein (MAP) kinase group which plays a crucial role in multiple signaling cascades, including the NF-κB pathway [25]. The expression of MLK3 was upregulated in tumor tissues and was associated with tumor progression and metastasis [26]. Based on our previous work, we designed a sponge model including SNHG12 and miR-199a/b-5p in HCC (Fig. 4e and f).

## Conclusion

In conclusion, the current study revealed that SNHG12 may be required for tumorigenesis and has the potential to be developed as a clinically promising biomarker for malignant phenotype of HCC. Furthermore, SNHG12 was found to function as a sponge for miR-199a/b-5p to reduce the inhibiting effect on MLK3, and thus enhanced the expressions of MLK3 and its downstream effectors in the NF-κB pathway.

## References

[1] El-Serag HB, Rudolph KL (2007). Hepatocellular carcinoma: epidemiology and molecular carcinogenesis. Gastroenterology.

[2] Lau WY, Lai EC (2008). Hepatocellular carcinoma: current management and recent advances. Hepatobiliary Pancreat Dis Int.

[3] Aravalli RN, Steer CJ, Cressman EN (2008). Molecular mechanisms of hepatocellular carcinoma. Hepatology.

[4] Shi X, Sun M, Liu H, Yao Y, Song Y (2013). Long non-coding RNAs: a new frontier in the study of human diseases. Cancer Lett.

[5] Huang S, He X (2011). The role of microRNAs in liver cancer progression. Br J Cancer.

[6] Mannoor K, Liao J, Jiang F (2012). Small nucleolar RNAs in cancer. Biochim Biophys Acta.

[7] Huang JL, Zheng L, Hu YW, Wang Q (2014). Characteristics of long non-coding RNA and its relation to hepatocellular carcinoma. Carcinogenesis.

[8] Fatica A, Bozzoni I (2014). Long non-coding RNAs: new players in cell differentiation and development. Nat Rev Genet.

[9] Gupta RA, Shah N, Wang KC (2010). Long non-coding RNA HOTAIR reprograms chromatin state to promote cancer metastasis. Nature.

[10] Tripathi V, Shen Z, Chakraborty A (2013). Long noncoding RNA MALAT1 controls cell cycle progression by regulating the expression of oncogenic transcription factor B-MYB. PLoS Genet.

[11] Ruan W, Wang P, Feng S, Xue Y, Li Y (2016). Long non-coding RNA small nucleolar RNA host gene 12 (SNHG12) promotes cell proliferation and migration by upregulating angiomotin gene expression in human osteosarcoma cells. Tumour Biol.

[12] Tay Y, Rinn J, Pandolfi PP (2014). The multilayered complexity of ceRNA crosstalk and competition. Nature.

[13] Tuo YL, Li XM, Luo J (2015). Long noncoding RNA UCA1 modulates breast cancer cell growth and apoptosis through decreasing tumor suppressive miR-143. Eur Rev Med Pharmacol Sci.

[14] Cao C, Zhang T, Zhang D, et al. The long non-coding RNA, SNHG6-003, functions as a competing endogenous RNA to promote the progression of hepatocellular carcinoma. Oncogene. 2016. doi:10.1038/onc.2016.278. 10.1038/onc.2016.278 27530352

[15] Zhou X, Ji G, Ke X, Gu H, Jin W, Zhang G (2015). MiR-141 Inhibits Gastric Cancer Proliferation by Interacting with Long Noncoding RNA MEG3 and Down-Regulating E2F3 Expression. Dig Dis Sci.

[16] Li H, Li J, Jia S (2015). miR675 upregulates long noncoding RNA H19 through activating EGR1 in human liver cancer. Oncotarget.

[17] Hou J, Lin L, Zhou W (2011). Identification of miRNomes in human liver and hepatocellular carcinoma reveals miR-199a/b-3p as therapeutic target for hepatocellular carcinoma. Cancer Cell.

[18] Mudduluru G, Ceppi P, Kumarswamy R, Scagliotti GV, Papotti M, Allgayer H (2011). Regulation of Axl receptor tyrosine kinase expression by miR-34a and miR-199a/b in solid cancer. Oncogene.

[19] Li SQ, Wang ZH, Mi XG, Liu L, Tan Y (2015). MiR-199a/b-3p suppresses migration and invasion of breast cancer cells by downregulating PAK4/MEK/ERK signaling pathway. IUBMB life.

[20] Song T, Zhang X, Yang G, Song Y, Cai W (2015). Decrement of miR-199a-5p contributes to the tumorigenesis of bladder urothelial carcinoma by regulating MLK3/NF-kappaB pathway. Am J Transl Res.

[21] Kunisada R, Yoshida N, Nakamura S, Uchiyama H, Matsumoto H. Enhanced expression of miR-199b-5p promotes proliferation of pancreatic beta-cells by down-regulation of MLK3. Microrna. 2016. 10.2174/2211536605666160607082214 27280801

[22] Gregory RI, Chendrimada TP, Cooch N, Shiekhattar R (2005). Human RISC couples microRNA biogenesis and posttranscriptional gene silencing. Cell.

[23] Spizzo R, Almeida MI, Colombatti A, Calin GA (2012). Long non-coding RNAs and cancer: a new frontier of translational research?. Oncogene.

[24] Iorio MV, Croce CM (2012). microRNA involvement in human cancer. Carcinogenesis.

[25] Hehner SP, Hofmann TG, Ushmorov A (2000). Mixed-lineage kinase 3 delivers CD3/CD28-derived signals into the IkappaB kinase complex. Mol Cell Biol.

[26] Chen J, Miller EM, Gallo KA (2010). MLK3 is critical for breast cancer cell migration and promotes a malignant phenotype in mammary epithelial cells. Oncogene.

